# Barrett's Esophageal Adenocarcinoma with Neuroendocrine Cell Carcinoma Treated by Endoscopic Submucosal Dissection: A Case Report and Literature Review

**DOI:** 10.1002/deo2.70167

**Published:** 2025-06-24

**Authors:** Yo Kubota, Kenji Ishido, Kusutaro Doi, Gen Kitahara, Takuya Wada, Akinori Watanabe, Hisatomo Ikehara, Chika Kusano

**Affiliations:** ^1^ Department of Gastroenterology Kitasato University School of Medicine Sagamihara Kanagawa Japan

**Keywords:** Barrett's esophageal adenocarcinoma, Barrett's esophagus, endoscopic submucosal dissection, neuroendocrine cell carcinoma, treatment

## Abstract

Barrett's esophageal adenocarcinoma (BEA) with neuroendocrine cell carcinoma (NEC) is a rare disease with a poor prognosis. We report a case of BEA with NEC treated by endoscopic submucosal dissection (ESD) with en bloc resection and long‐term survival was achieved without additional treatment. An 84‐year‐old man underwent an upper gastrointestinal endoscopy, which revealed an erythematous lesion within a short‐segment Barrett's esophagus (BE) at the esophagogastric junction. A diagnosis of well‐differentiated tubular adenocarcinoma (tub1) was made after biopsy, specifically, BEA (EG, Type 0‐IIa, 15 mm, cT1a), and ESD was performed. Histopathological findings showed differentiated adenocarcinoma in Barrett's esophageal mucosa background, with 20% of all tumors having NEC components. The final diagnosis was adenocarcinoma (tub1>tub2) with focal neuroendocrine cell carcinoma in BE, EG, Type 0‐IIa, 13 × 9 mm, pT1a‐DMM, ly0, v0 pHM0, pVM0. Considering the patient's advanced age and wishes, we decided to follow up without any additional treatment. The patient has survived 52 months after ESD without metastatic recurrence. Even if histopathological findings after ESD reveal BEA with NEC and en bloc resection, careful follow‐up is necessary because of the high risk of recurrence.

## 1 Introduction

The incidence of Barrett's esophageal adenocarcinoma (BEA) has increased in recent years, especially in Western countries [1]. However, BEA with esophageal neuroendocrine cell carcinoma (NEC) is very rare. Endoscopic therapy, surgery, and chemotherapy are considered for treatment [2, 3, 4], although the disease progresses rapidly and has a poor prognosis with an overall survival of 14 months [5]. Here, we report a case of BEA with NEC that was resected en bloc through endoscopic submucosal dissection (ESD); notably, the patient has survived for 52 months without additional treatment.

## 2 Case Report

An 84‐year‐old man was referred to our hospital for unexpected weight loss (3 kg in 2 months). He had a history of diabetes, hypertension, and hyperlipidemia, smoked 20 cigarettes/day for 44 years, and had never consumed alcohol. He was taking oral hypoglycemic, antihypertensive, and lipid‐regulating drugs but no antithrombotic drugs or proton pump inhibitors. Physical examination revealed no significant findings.

**FIGURE 1:**
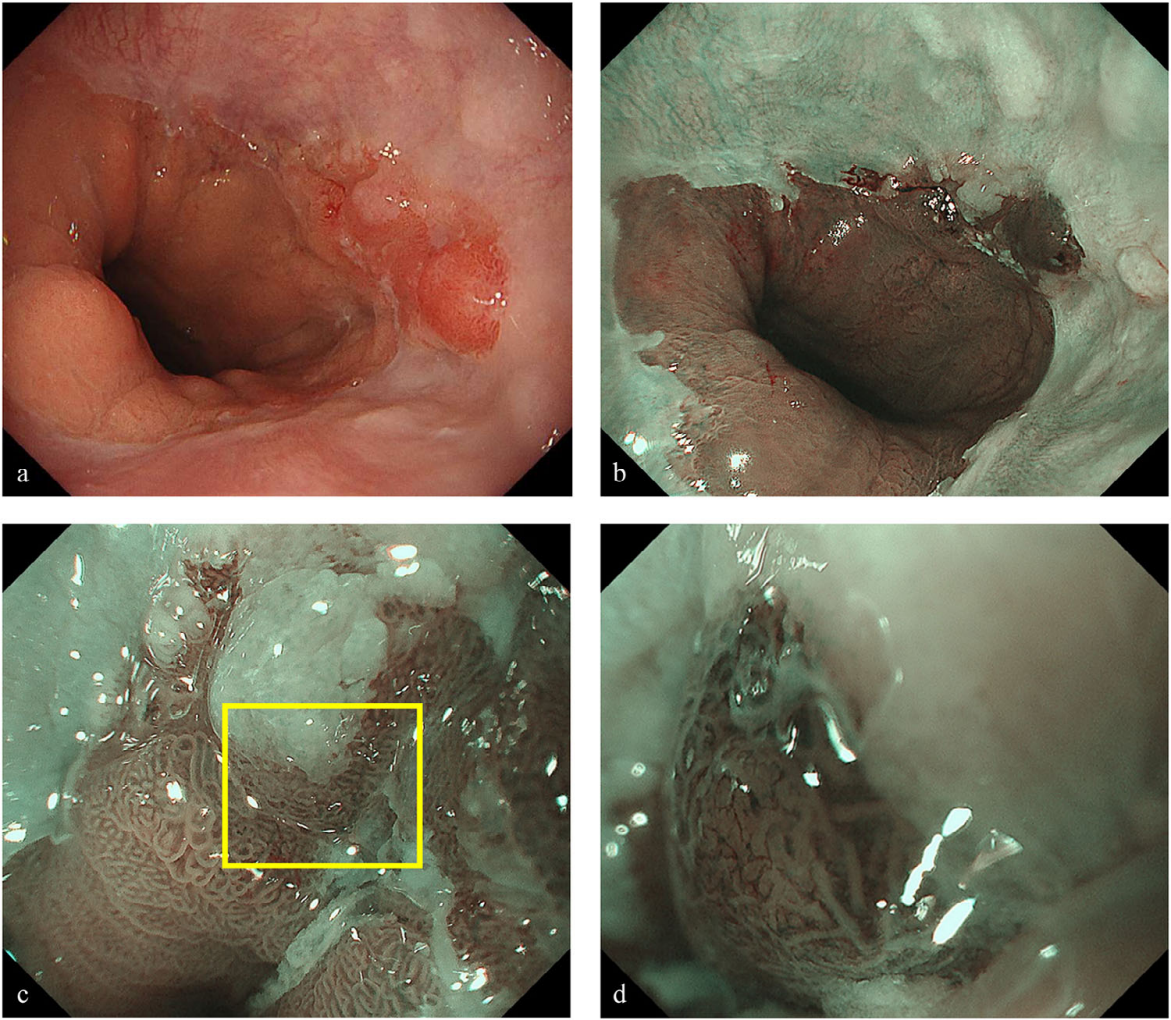
Barrett's esophageal adenocarcinoma at diagnosis. (a) Upper gastrointestinal endoscopy showing the 15‐mm erythematous flat elevated lesion in the esophagogastric junction at 3 o'clock. (b) Narrow band imaging (NBI): Lesions are recognized as brownish areas. (c) NBI‐magnifying endoscopy: Demarcation line, irregular microsurface pattern, and irregular microvascular pattern (yellow square) are noted. (d) When the lesion is enlarged, an irregular microvascular pattern is observed, which suggests a neoplastic lesion.

Upper gastrointestinal endoscopy under white light imaging revealed palisade vessels from the 12 to 1 o'clock direction at the gastroesophageal junction, leading to a diagnosis of short‐segment Barrett's esophagus (BE). In addition, a 15‐mm erythematous, flat elevated lesion with irregular margins was identified at the 3 o'clock position of the gastroesophageal junction (Figure 1a). Narrow‐band imaging (NBI) identified the lesion as a brownish area (Figure 1b). NBI‐magnifying endoscopy revealed a demarcation line, irregular microsurface pattern, and irregular microvascular pattern (Figure 1c,d), indicative of a neoplastic lesion. The tumor was extended by air delivery, and the depth of the lesion was considered equivalent to that of T1a. Biopsy results showed a well‐differentiated tubular adenocarcinoma (tub1). Computed tomography (CT) revealed no evidence of metastatic lesions in the lymph nodes and distant organs. Based on these findings, a diagnosis of BEA (EG, Type 0‐IIa, 15 mm, cT1a) was made, and ESD was performed using an IT‐knife nano device (Olympus Medical Systems Co., Ltd, Tokyo, Japan). The procedure was completed without any complications.

**FIGURE 2:**
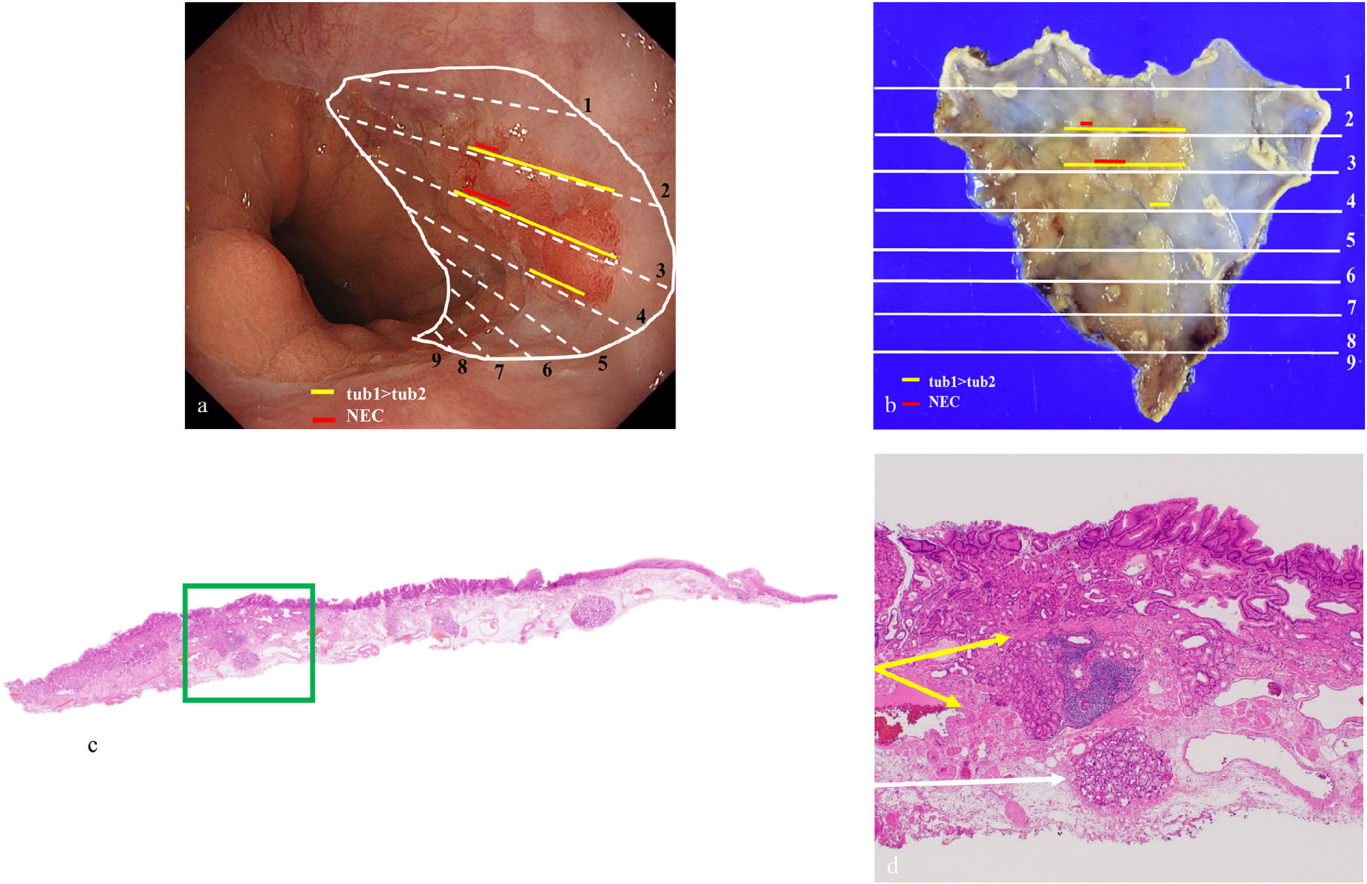
Pathological examination. (a) Mapping of lesions in endoscopic findings. Barrett's esophageal adenocarcinoma (BEA) is indicated by the yellow line, and NEC by the red line. (b) Mapping of lesions in resected specimens. BEA is indicated by the yellow line, and NEC by the red line. (c) Pathological mapping on line 7 of the resected specimens. BE is indicated by the green square (hematoxylin and eosin staining). (d) Magnified view of the BE (green square in Figure 2c). The intrinsic esophageal glands (white arrows) and the double layer of muscularis mucosae under the columnar epithelium (yellow arrows) were visible.

**FIGURE 3:**
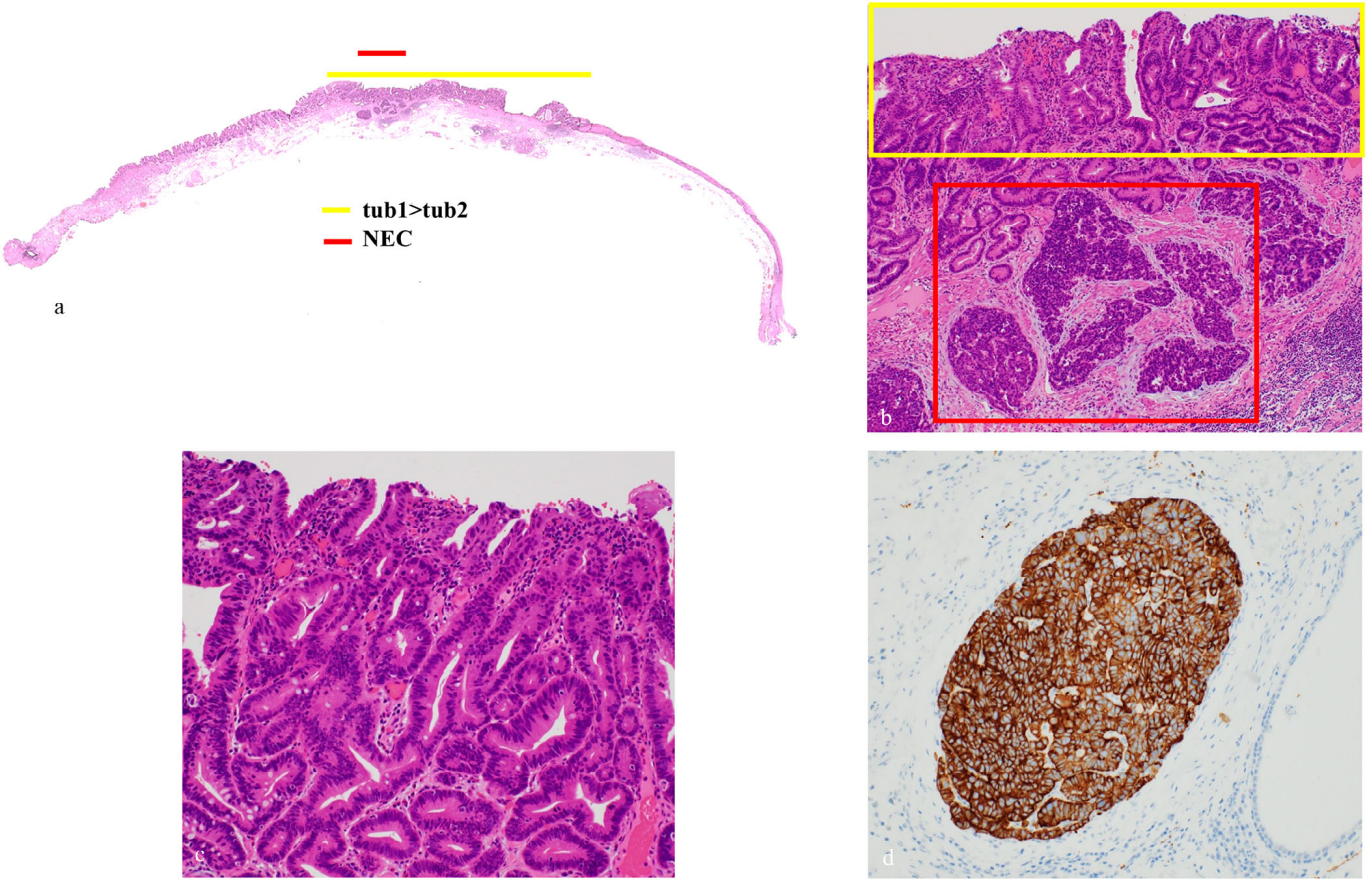
Pathological examination (Barrett's esophageal adenocarcinoma and Neuroendocrine cell carcinoma). (a) Pathological mapping on line 3 of the resected specimens. Barrett's esophageal adenocarcinoma (BEA) is indicated by the yellow line, and NEC by the red line (hematoxylin and eosin staining). (b) BEA is indicated by the yellow square. Within the deeper portion of the BEA, dense aggregates of atypical cells with a high nuclear/cytoplasmic ratio are visible from the proper mucosal layer to the lamina muscularis mucosae (red square) (hematoxylin and eosin staining, ×40 magnification). (c) Magnified view of the BEA (yellow square in Figure 3b) (hematoxylin and eosin staining, ×200 magnification). (d) Synaptophysin positive (red square in Figure 3b) (hematoxylin and eosin staining, ×200 magnification).

Histopathological findings are shown (Figure 2a,b). There was a double layer of muscularis mucosae under the columnar epithelium, with BE in the background (Figure 2c,d). The lesion showed a well‐differentiated to moderately differentiated tubular adenocarcinoma that grew in an irregular tubular structure from the mucosal epithelium to the proper mucosal layer and partially to the lamina muscularis mucosae, consistent with a raised area (Figure 3a–c). Furthermore, atypical cells with a high nuclear/cytoplasmic ratio proliferated densely spanning from the proper mucosal layer to the lamina muscularis mucosae (Figure 3b). These cells were positive for chromogranin A and synaptophysin (Figure 3d) and had a mitotic rate of 2–3 per 10 high‐power fields (HPFs), and a Ki67 index of 20–30%, which led to the diagnosis of NEC. As we measured the area occupied by the NEC on histological sections and calculated its proportion relative to the total tumor area, which was found to be 20%, the final diagnosis was BEA (tub1> tub2) with focal neuroendocrine carcinoma, EG, Type 0–IIa, 13×9 mm, pT1a‐DMM, ly0, v0, pHM0, pVM0. After the endoscopic treatment, the patient was discharged without significant adverse events. Considering the patient's advanced age and wishes, no additional surgery or adjuvant chemotherapy was performed. The risk of recurrence was fully explained to the patient. His progress was carefully monitored with an upper gastrointestinal endoscopy and a CT every 6 months. At the time of writing, the patient has survived for 52 months after ESD without metastatic recurrence.

## 3 Discussion

BE is characterized by a change from esophageal squamous epithelium to columnar epithelium with goblet cells and is recognized as a precancerous lesion of BEA [6]. BEA incidence is estimated to be 1.1% of all esophageal cancers [1]. However, its incidence has increased in recent years, mainly in Western countries. In contrast, NEC is a rare malignancy, accounting for only 0.3% of all esophageal cancers [1]. NEC onset is reported to be preceded by BEA, followed by clonal differentiation of a pluripotent cell into a neuroendocrine phenotype [7]. Kinoshita et al. reported that adenocarcinoma and NEC intermingle in the transition zone, with continuity observed in both lesions and that NEC may be a neuroendocrine differentiation of BEA [2]. Conversely, Spagnolo et al. proposed the diagnosis of collision carcinomas, in which two distinct histologic types are clearly distributed, and each can be clearly recognized at the border between tumors [8]; however, the exact mechanism remains unclear. In this case, the BEA and NEC were vertically aligned with a distinct vertical boundary but without horizontal contact, suggesting the possibility of a vertically arranged composite tumor.

As per WHO classification, neuroendocrine tumor (NET) is classified as NET G1 (well‐differentiated, mitotic rate < HPFs and/or Ki67 index < 3%), NET G2 (well‐differentiated, mitotic rate 2–20 HPFs and/or Ki67 index 3–20%), G3 (well‐differentiated, mitotic rate > 20 HPFs and/or Ki67 index > 20%), NEC (Poorly differentiated, mitotic rate > 20 HPFs and/or Ki67 index > 20%), and mixed neuroendocrine‐non‐neuroendocrine neoplasm (MiNEN) [9]. MiNEN is characterized by NET and non‐NET components, each occupying >30% of the tumor; the non‐NET component is an invasive carcinoma, and NET and non‐NET are composite tumors that migrate and mix [9]. Immunostaining images are also useful in NET diagnosis, and neuroendocrine markers such as chromogranin A and synaptophysin are expressed in 69.1% and 90.2% of NET, respectively [10]. In the present case, samples were positive for chromogranin A and synaptophysin, and had a Ki67 index of 20%–30%, but could not be categorized as having MiNEN because NEC accounted for only 20% of the total tumor.

Several studies have reported BEA with NEC components confined to the T1 layer (Table 1). Preoperative biopsies, including that of this case, predominantly reveal adenocarcinomas, making diagnosis challenging because of the tendency of NEC to develop subepithelial growth. NEC has a poor prognosis owing to its rapid progression and high risk of metastasis and recurrence; therefore, additional surgery and adjuvant chemotherapy [Cisplatin + Etoposide (VP‐16) or Irinotecan (CPT‐11)] may be considered even if the lesion is subjected to endoscopic resection [10]. Even if the lesions are localized and resectable at the time of surgery, multimodal treatment may improve the prognosis of esophageal NEC. For instance, Bibeau et al. reported a case of long‐term survival after combined chemoradiotherapy and surgery for locally advanced BEA with NEC [4].

**TABLE 1:** Previously reported cases of neuroendocrine cell carcinoma concomitant with Barrett's esophageal adenocarcinoma (only T1 lesions).

Author(s)	Age/sex	Pretreatment biopsy	Treatment	Tumor size (mm)	Depth	Lymph node metastasis	Adjuvant chemotherapy	Recurrence	Observation period
Slavin et al.	63/female	Adenocarcinoma	Surgery	50	T1	N1	Not provided	N.E.	3 months
Wilson et al.	51/male	Adenocarcinoma	Surgery	40	T1b	N0	Provided	N.E.	N.E.
Doi et al.	71/male	Adenocarcinoma	Surgery	10	T1b	N0	Provided	None	36 months
Kawazoe et al.	70/male	Adenocarcinoma with NEC	Surgery	25	T1	N0	Not provided	None	4 months
Kinoshita et al.	79/male	Adenocarcinoma	ESD/ surgery	10	T1b	N0	Not provided	None	48 months
Vetis et al.	68/male	Adenocarcinoma	ESD	N.E.	T1b	N.E.	N.E.	N.E.	N.E.
Present Case	84/male	Adenocarcinoma	ESD	13	T1a‐DMM	N0	None	None	52 months

Abbreviations: ESD, endoscopic submucosal dissection; N.E., not evaluated; NEC, Neuroendocrine cell carcinoma.

To the best of our knowledge, this is the first report of BEA with NEC where long‐term survival of 52 months was achieved with ESD resection alone. Several factors may have contributed to the long‐term survival of the patient with ESD alone, including the size of the lesion (13 mm), that only 20% of the total tumor was NEC, that the NEC was limited to lamina muscularis mucosae, and that en bloc resection was completed successfully. Nevertheless, this is not an established treatment protocol for such cases, and careful follow‐up using upper gastrointestinal endoscopy and CT is necessary to monitor the risk of metastatic recurrence. Moreover, it is desirable to establish a treatment strategy by accumulating more cases in the future.

In conclusion, if pathology after endoscopic resection reveals BEA with NEC, careful follow‐up is necessary after fully explaining to the patient the need for additional surgery or adjuvant chemotherapy, considering the risk of metastatic recurrence.

## Consent

Informed consent was obtained from the patient for publication of this report and accompanying images.

## Conflicts of Interest

The authors declare no conflicts of interest.
